# 

**Published:** 1856-04

**Authors:** 


Wanted.—Ths senior editor wishes to purchase the ***first volume*** of the first, or old series of the Journal, for which he will give $5 00. He wants No 1, of vol. 2; No. 3, of vol. 7, and No 1, of vol. 9, for which he will give $1 25 a number. He will also pay the same price for No. 1, vol. 1, and No. 2, vol. 2, of the new series.

